# Harmful alcohol drinking among HIV-positive people in Nepal: an overlooked threat to anti-retroviral therapy adherence and health-related quality of life

**DOI:** 10.1080/16549716.2018.1441783

**Published:** 2018-03-02

**Authors:** Khem Narayan Pokhrel, Kalpana Gaulee Pokhrel, Sanjeev Raj Neupane, Vidya Dev Sharma

**Affiliations:** ^a^ Department of HIV, Nutrition, and Health, Health Research and Social Development Forum, Kathmandu, Nepal; ^b^ Department of HIV and Nutrition, Integrated Development Foundation, Kathmandu, Nepal; ^c^ Department of Psychiatry and Mental Health, Institute of Medicine, Tribhuwan University, Kathmandu, Nepal

**Keywords:** HIV, harmful alcohol drinking, anti-retroviral therapy, quality of life, Nepal

## Abstract

**Background**: People living with Human Immunodeficiency Virus (HIV) often suffer from alcohol-use disorders resulting in their poor health and treatment outcomes. Little is known about the association of harmful alcohol drinking with their adherence to anti-retroviral therapy (ART) and health-related quality of life (QOL) in low-resource settings.

**Objective**: This study aimed to investigate associations between harmful alcohol drinking, adherence to ART and health-related QOL in HIV-positive people, stratified by gender, in Nepal.

**Methods**: We conducted a cross-sectional study of 682 HIV-positive people on ART to measure their self-reported harmful alcohol drinking and non-adherence to ART in the previous month of data collection. We also measured health-related QOL using a WHOQOL-HIV BREF scale. The association between harmful alcohol drinking and non-adherence to ART was examined using multiple logistic regressions. Additionally, multiple linear regressions examined association between harmful alcohol drinking and QOL.

**Results**: Harmful alcohol drinking was associated with non-adherence to ART among men (AOR: 2.48, 95% CI: 1.50, 4.11, p < 0.001) and women (AOR: 2.52, 95% CI: 1.32, 4.80, p = 0.005). Men were more likely to have lower score for the psychological (β = −0.55, p = 0.021) and level of independence (β = −0.68, p = 0.018) domains when they had harmful alcohol drinking. Moreover, women were more likely to have lower scores for the physical (β = −1.01, p = 0.015), social relations (β = −0.82, p = 0.033), environmental (β = −0.88, p = 0.011), and spiritual (β = −1.30, p = 0.005) domains of QOL when they had harmful alcohol drinking.

**Conclusions**: Harmful alcohol drinking had a negative association with ART adherence and QOL in both HIV-positive men and women in Nepal. Screening for alcohol-use disorders and community-based counseling services should be provided while delivering ART services to improve treatment adherence and QOL.

## Background

Access to anti-retroviral therapy (ART) improved the life expectancies of people living with Human Immunodeficiency Virus (HIV) globally [1,2]. However, their medication adherence remains salient. Many suffer from harmful alcohol drinking disorders, this being one of the common behavioral disorders [3]. In HIV-positive people, alcohol consumption increases the risk of HIV disease progression, leading to poor health outcomes [4]. Harmful alcohol drinking eventually results in negative consequences for medication adherence [5] and poor health-related quality of life (QOL) [6].

Harmful alcohol drinking among HIV-positive people has been associated with non-adherence to ART in both high-income countries (HICs) and in low- and middle-income countries (LMICs) [7–9]. In a study in Peru, harmful alcohol drinking had inverse effects on ART adherence among men who have sex with men (MSM) [10]. In this study, 31.9% of the HIV-positive people practiced drinking alcohol at a harmful level, as measured by the alcohol-use disorders identification test (AUDIT). In the United States, HIV-positive people experienced poor social and emotional support from family and friends when they continued consuming alcohol. Such poor support has been shown to have a negative impact on adherence to ART [11]. In addition, HIV-positive people who use alcohol frequently can experience more conflict with their partners, which can impact negatively on spousal support and lead to poor compliance with ART [12]. Harmful alcohol drinking can also reduce coping skills and increase the likelihood of opportunistic infections [13]. Moreover, HIV-positive people who used illicit substances also had alcohol-use disorders resulting in non-adherence to ART [14].

People living with HIV who use alcohol tend to have a poor health-related QOL. Research in the United States shows that alcohol-use disorders can impact negatively on physical functioning and on the emotional well-being of HIV-positive people, which can then decrease mental health-related QOL [15]. In that study, HIV-positive people who suffered from alcohol-use disorders were less likely to have perceived social support, which resulted in poor QOL in the psychological domain. Moreover, another study has shown that HIV-positive people who usually did not seek social support, experienced poor psychological QOL [16]. Alcohol use also increases the risk of substance use such as heroin, which may have deleterious effects on health-related QOL in both physical and mental health dimensions [5,17].

Gender difference also has implications in relation to the association between alcohol use and health outcomes. In the United States, men having sex with men (MSM) had a higher prevalence of HIV because of unprotected sexual practices resulting from harmful alcohol drinking behavior [18]. Women in the United States also tended to receive poor social support, which had negative effects on adherence to ART when they suffered from alcohol-use disorders [19].

HIV-positive people who use alcohol, poorly engage in HIV services and this affects their continuum of care. A systematic review found that alcohol users were more likely to have missed at least one or more cascades of continuum of care such as attending treatment services, ART compliance, routine investigation of viral loads, and CD4 count [20]. Additionally, alcohol users also engage in substance use as a coping mechanism for reducing stress related to HIV, which further deteriorates their health [21].

HIV-positive people had non-adherence to ART when they consumed alcohol in Nepal [22,23]. Alcohol consumption, however, was measured using a single-item scale in Nepal to examine the association of alcohol use and non-adherence using AIDS Clinical Trial Group (ACTG) questionnaires. Little is known about the harmful effects of drinking alcohol and its association with ART adherence in Nepal. A measure that is more comprehensive would be helpful in covering harmful alcohol drinking that comprises dose, frequency, and hazardous alcohol use. Alcohol use is common in both rural and urban areas of the country because homemade alcohol and brewed alcohol are easily available [24]. Additionally, no evidence is available about the role of harmful alcohol drinking on the QOL of HIV-positive people stratified by gender in South Asia. This study was conducted on HIV-positive people in Nepal. The aims were, first, to investigate associations between harmful alcohol drinking and adherence to ART among HIV-positive people stratified by gender, and, second, to examine the association between harmful alcohol drinking and health-related QOL in HIV-positive people stratified by gender.

## Methods

### Study setting

This study was conducted in Nepal, a low-income country situated in South Asia, which harbors a population of 28 million. About 40,000 HIV-positive people are living in the country. Among them, approximately 12,000 were on ART in 2015 [25].

### Study design and participants

The design was cross-sectional. The study districts were selected by convenience sampling method and participants were conveniently recruited from the various non-governmental organizations (NGOs) involved in providing HIV services. The participants who took ART for at least one year at the time of data collection and those who were diagnosed as HIV-positive within five years were included in the study sample.

### Sampling procedures

We calculated the sample size using G*Power statistical software. First, we took the reference prevalence of non-adherence to ART as 21% among HIV-positive people who received community-based psychosocial support [26]. Second, a previous study found a 10% difference in non-adherence to ART among HIV-positive people who received psychosocial support compared to those who did not receive the support [27]. We assumed the same differences of 10% non-adherence to ART between participants who would enroll in a community home-based care program and those who would not enroll in the program. This resulted in an estimated prevalence of non-adherence to ART among the control group as 31%. With p-value for significant of less than 0.05 (two-tailed) and the power of 0.80, the calculated sample size was 638 (intervention: 319; control: 319). In total, we recruited 720 participants, of them, 682 were considered in the analysis.

## Measures

### Harmful alcohol drinking

Participants were asked about their regular drinking behavior. The extent of harmful drinking was measured using the Alcohol Use Disorders Identification Test (AUDIT), which is a 10-item scale designed to measure alcohol use covering amount, frequency, and hazardous drinking [10,28]. The available alcoholic drinks such as locally brewed alcohol; bottled wine, beer, whisky, vodka, etc. were standardized as one unit for each 10 gm of alcohol content. For example, if an individual consumed 200 ml of locally brewed alcohol with concentration of 25% it implies that he/she consumed five units of alcohol. The scale has already been validated and applied in Nepal [29], and the Cronbach’s alpha was 0.78 in this study. A cumulative total score ranges from 0 to 40. Scores of eight or more were categorized as being harmful alcohol drinking.

### ART non-adherence

The questionnaires developed by the AIDS Clinical Trial Group (ACTG) were applied to assess the missed pills. The data collection considered participants who missed at least one dose in the past month as being non-adherent to ART [30,31].

### Health-related QOL

Participants’ health-related QOL was measured using the WHO Quality of Life-HIV BREF (WHOQOL-HIV BREF) scale [32]. The scale measures physical, psychological, level of independence, social relations, environmental, and spiritual/religious domains of life. Each item score ranges from 1 to 5 on a Likert scale. A score of ‘1’ indicates a low and negative direction and ‘5’ indicates a high and positive direction. Six domains of QOL were calculated using various facets of the scale. The scores were arranged reversely, which had a negative direction (pain and discomfort, negative feelings, dependence on medication, death and dying). The total scores for each domain range from 4 to 20. Higher scores indicate a better QOL. The values of Cronbach’s alpha for the domains were: physical 0.78; psychological 0.84; level of independence 0.82; social relations 0.86; environmental 0.88; and spiritual/religion 0.83. The scale was developed by World Health Organization (WHO) and it has been widely used in various countries including Nepal [32–34].

### Socio-demographics

Participants were interviewed and asked for self-reported information regarding age, gender, marital status education, employment status, and physical symptoms. Marital status was categorized as married and unmarried/widow/single. Education level was grouped in two categories: illiterate and primary level education; or more. For employment status, we categorized the participants as employed/self-employed and unemployed. Further participants who reported opportunistic infections were categorized as having physical symptoms and we considered those who did not report any symptoms related to opportunistic infection as having no physical symptoms.

### HIV-clinical staging

The records about clinical staging were retrieved from the ART centers of the selected districts. The first and second clinical stages were categorized as early stage, and the third and fourth stages were categorized as advanced [35].

### Procedure for data collection

A set of questionnaires was first translated from English to Nepali and then back translated to English by two independent translators, to ensure consistency in meaning and interpretation. Finally, some modifications were made before finalizing the questionnaires for pre-testing. Pre-testing of questionnaires was done among 48 participants in Kathmandu. Necessary modifications were made in the questionnaire after the pre-testing. We approached 720 HIV-positive people on ART and 682 participants (94.7%) responded to the questions. The pre-tested participants were not included in the study. The baseline data collection was conducted in February and March 2015.

### Data analysis

Data were analyzed descriptively using chi-squared and Fisher’s exact test for categorical variables and a t-test for age. Multiple logistic regressions examined the association between harmful alcohol drinking and non-adherence to ART. Additionally, multiple linear regressions examined the association between harmful alcohol drinking and the quality of life (QOL) domains. In each model, important covariates such as age, education, marital status, occupation, physical symptoms, and HIV-clinical staging were adjusted, as studies have indicated that these factors can influence ART adherence [30,31]. All data were analyzed using STATA version 12.1.

### Ethical considerations

The study was approved by the Research Ethics Committee of the Graduate School of Medicine, the University of Tokyo and the Nepal Health Research Council. Prior to data collection, permission was obtained from the selected agencies providing community home-based care and support services for HIV-positive people in Nepal. Participants were ensured about the confidentially of information and their voluntary participation. Moreover, written informed consent was obtained from the participants on a designated form. The participants were also asked for their thumbprint if they were not able to write their name. During the interview, if participants were found to have a severe alcohol-use disorder, they were referred to non-governmental organizations and nearby hospitals.

## Results

### Socio-demographic characteristics and harmful alcohol drinking of participants

Of 682 participants, 354 were men and 328 were women. About 25.1% of the men and 29.3% of the women were non-adherent to ART. ART adherent and non-adherent participants were not significantly differences in relation to their age, education, marital status, employment, HIV-clinical staging, or physical symptoms. Among them, 35.0% of the men and 15.5% of the women had harmful alcohol drinking. Compared with adherent men, more non-adherent men had harmful alcohol drinking (51.1% vs. 29.6%, p < 0.001). A significantly higher proportion of non-adherent women had harmful alcohol drinking compared to those who were ART adherent (22.7% vs. 12.5%, p = 0.013) (see Table 1).

**Table 1.** Sociodemographic characteristics and harmful alcohol drinking among ART adherent and non-adherent participants.

		Men (n = 354)			Women (n = 328)		
Variables	Total n (%)	ART adherent (n = 265) n (%)	ART non- adherent (n = 89) n (%)	P-value	ART adherent (n = 232) n (%)	ART non-adherent (n = 96) n (%)	P-value
**Age (mean, SD)^a^**	36.3 (8.2)	36.9 (8.8)	36.7 (7.8)	0.845	35.5 (8.1)	36.1 (8.0)	0.893
**Education level^b^**							
Illiterate	289 (42.4)	91 (34.3)	36 (40.4)	0.265	111 (47.8)	51 (53.1)	0.384
≥Primary level	393 (57.6)	174 (65.7)	53 (59.6)		121 (52.2)	45 (46.9)	
**Marital status^b^**							
Married	554 (81.2)	225 (84.9)	64 (71.9)	0.016	189 (81.4)	76 (79.2)	0.828
Unmarried/widow/single	128 (18.8)	40 (15.1)	25 (28.1)		43 (18.6)	20 (20.8)	
**Employment status^b^**							
Employed/self-employed	448 (65.7)	147 (55.5)	63 (70.8)	0.060	162 (69.8)	76 (79.2)	0.085
Unemployed	234 (34.3)	118 (44.5)	26 (29.2)		70 (30.2)	20 (20.8)	
**HIV-clinical staging^b^**							
Early stage	550 (80.6)	219 (86.6)	67 (77.0)	0.039	192 (84.6)	72 (75.8)	0.062
Advanced stage	132 (19.4)	34 (13.4)	20 (23.0)		35 (15.4)	23 (24.2)	
**Physical symptoms^b^**							
Yes	146 (21.4)	44 (16.6)	23 (25.8)	0.050	52 (22.4)	27 (28.1)	0.271
No	536 (78.6)	221 (83.4)	66 (74.2)		180 (76.6)	69 (71.9)	
**Harmful alcohol drinking^b^**							
Yes	175 (25.7)	78 (29.6)	46 (51.1)	<0.001	29 (12.5)	22 (22.7)	0.013
No	507 (74.3)	186 (70.4)	44 (48.9)		202 (87.5)	75 (77.3)	

^a^Independent sample t-test.

^b^Chi-squared test.

### Multiple logistic regression: association of harmful alcohol drinking with non-adherence to ART stratified by gender

Men were more likely to be non-adherent to ART when they had harmful alcohol drinking (AOR: 2.48, 95% CI: 1.50, 4.11, p < 0.001). Also, harmful alcohol drinking was positively associated with non-adherence to ART among women (AOR: 2.52, 95% CI: 1.32, 4.80, p = 0.001) (see Table 2).

**Table 2.** Logistic regression analysis: the association between harmful alcohol drinking and non-adherence to ART stratified by gender.

	ART non-adherence			
	Men		Women	
Characteristics	AOR^a^ (95% CI)	P-value	AOR^a^ (95% CI)	P-value
Harmful alcohol drinking	2.48 (1.50, 4.11)	<0.001	2.52 (1.32, 4.80)	0.005
Marital status (married)	1.00 (0.96, 1.03)	0.964	0.99 (0.96, 1.02)	0.696
Age	0.95 (0.56, 1.62)	0.862	0.82 (0.49, 1.37)	0.842
Education (≤primary)	0.98 (0.57, 1.69)	0.962	0.76 (0.46, 1.26)	0.298
Employment status (employed/self-employed)	0.53 (0.27, 1.04)	0.067	0.83 (0.44, 1.57)	0.580
HIV-clinical staging (advanced)	0.56 (0.32, 0.99)	0.047	0.69 (0.38, 1.26)	0.239
Physical symptoms (present)	1.23 (0.41, 3.66)	0.707	0.79 (0.29, 2.13)	0.653

^a^Adjusted odds Ratio.

### Harmful alcohol drinking by QOL domains

Compared to those who did not have harmful alcohol drinking, men had lower scores for psychological (mean: 12.3 vs. 12.9, p = 0.013), level of independence (mean: 12.0 vs. 12.8, p = 0.008), environmental (mean: 11.6 vs. 12.2, p = 0.010), and spiritual (mean: 11.1 vs. 12.1, p = 0.001) domains when they had harmful alcohol drinking.

Similarly, women who had harmful alcohol drinking had lower scores of physical (mean: 12.3 vs. 13.1, p = 0.041) and spiritual (mean: 10.6 vs. 11.7, p < 0.001) domains compared to those who did not have harmful alcohol drinking (see Table 3).

**Table 3.** Descriptive analysis: harmful alcohol drinking by QOL domains stratified by gender.

	Physical				Psychological				Level of independence			
	Men		Women		Men		Women		Men		Women	
Variable	Mean (SD)	P-value	Mean (SD)	P-value	Mean (SD)	P-value	Mean (SD)	P-value	Mean (SD)	P-value	Mean (SD)	P-value
HAD^a^												
No	13.1 (2.7)	0.091	13.1 (2.7)	0.041	12.9 (2.1)	0.013	12.5 (2.2)	0.577	12.8 (2.6)	0.008	12.4 (2.6)	0.274
Yes	12.6 (2.8)		12.3 (2.6)		12.3 (2.2)		12.3 (2.2)		12.0 (2.5)		12.0 (2.6)	
	Social relation				Environmental				Spiritual			
	Men		Women		Men		Women		Men		Women	
Variable	Mean (SD)	P-value	Mean (SD)	P-value	Mean (SD)	P-value	Mean (SD)	P-value	Mean (SD)	P-value	Mean (SD)	P-value
HAD^a^												
No	12.5 (2.5)	0.235	12.1 (2.8)	0.136	11.6 (2.1)	0.010	11.3 (2.3)	0.062	11.1 (3.0)	0.001	10.6 (2.6)	<0.001
Yes	12.8 (2.4)		12.6 (2.4)		12.2 (2.1)		11.9 (2.2)		12.1 (2.9)		11.7 (3.0)	

HAD = Harmful alcohol drinking.

^a^Independent sample t-test.

### Multiple linear regression: association of harmful alcohol drinking and QOL domains stratified by gender

After controlling age, marital status, education level, employment status, physical symptoms, and HIV-clinical staging, harmful alcohol drinking was negatively associated with QOL domains. Among men, harmful alcohol drinking was associated with the psychological (β = −0.55, p = 0.021), level of independence (β = −0.68, p = 0.018), environmental (β = −0.55, p = 0.022), and spiritual (β = −0.98, p = 0.004) domains. Additionally, women were more likely to have lower scores for physical (β = −1.01, p = 0.015), social relations (β = −0.82, p = 0.033), environmental (β = −0.88, p = 0.011), and spiritual (β = −1.30, p = 0.005) domains when they had harmful alcohol drinking (see Tables 4 and 5).

**Table 4.** Multiple regression analysis: the association of harmful alcohol drinking with the QOL domains (physical, psychological, and level of independence) stratified by gender.

	Physical				Psychological				Level of Independence			
	Men		Women		Men		Women		Men		Women	
Variables	β	SE	β	SE	β	SE	β	SE	β	SE	β	SE
Harmful alcohol drinking	−0.41	0.31	−1.01*	0.41	−0.55*	0.24	−0.39	0.34	−0.68*	0.29	−0.63	0.40
Marital status (married)	0.12	0.42	−0.13	0.38	0.01	0.32	−0.09	0.31	0.31	0.39	−0.29	0.37
Age	0.02	0.02	0.04*	0.02	0.02	0.02	0.04*	0.02	0.00	0.02	0.05	0.02
Education (≥primary)	0.89**	0.31	0.20	0.30	0.54	0.24	0.25	0.25	0.17	0.29	0.11	0.29
Employment status (Employed/self-employed)	−0.19	0.31	0.24	0.34	0.50*	0.24	0.12	0.28	0.10	0.29	0.24	0.33
Clinical stage (advanced)	−0.45	0.72	−1.53*	0.62	−0.35	0.55	−1.20*	0.51	−0.17	0.68	−0.64	0.60
Physical symptoms (present)	−0.54	0.68	0.34	0.55	−0.26	0.52	0.23	0.45	−0.70	0.64	−0.23	0.53

*p-value<0.05, **p-value<0.01.

**Table 5.** Multiple regression analysis: the association of harmful alcohol drinking with QOL domains (physical, psychological, and level of independence) stratified by gender.

	Social relation				Environmental				Spiritual			
	Men		Women		Men		Women		Men		Women	
Variables	β	SE	β	SE	β	SE	Β	SE	β	SE	β	SE
Harmful alcohol drinking	−0.28	0.28	−0.82*	0.38	−0.55*	0.24	−0.88*	0.34	−0.98**	0.33	−1.30**	0.46
Marital status (married)	0.11	0.37	0.03	0.36	0.22	0.32	−0.04	0.32	0.07	0.45	−0.09	0.43
Age	0.02	0.02	0.05	0.02	−0.01	0.02	0.03	0.02	0.01	0.02	−0.01	0.02
Education (≥primary)	0.20	0.28	0.05	0.28	0.18	0.24	−0.14	0.25	0.57	0.34	0.09	0.34
Employment status (Employed/self-employed)	0.30	0.28	0.41	0.31	0.18	0.24	0.26	0.28	0.39	0.34	−0.04	0.38
Clinical stage (advanced)	−0.68	0.65	−1.18*	0.57	−0.42	0.56	−1.42**	0.52	−0.89*	0.78	−1.28	0.69
Physical symptoms (present)	−0.04	0.61	0.38	0.51	−0.23	0.52	0.31	0.46	0.37	0.74	0.74	0.62

*p-value <0.05, **p-value <0.001.

## Discussion

In this study, HIV-positive men and women in Nepal were more likely to be non-adherent to ART when they had harmful alcohol drinking. Additionally, harmful alcohol drinking was negatively associated with the psychological, level of independence, environmental, and spiritual domains of QOL among men. Women were more likely to have poor QOL for physical, social relations, environmental, and spiritual domains when they had harmful alcohol drinking.

HIV-positive men and women who had harmful alcohol drinking were more likely to be non-adherent to ART. With regard to gender differences, both men and women had non-adherence. The strength of the association in women was slightly higher compared to men. In the Nepali context, alcohol drinking behavior might have impaired women to access to HIV services compared to men. It is possible that both men and women might not have sought family support and might have experienced poor coping skills because they suffered from other mental health and substance-use disorders such as use of heroin and cocaine.

Higher frequency of alcohol consumption induces a stronger maladaptive coping strategy among HIV-positive people because the alcohol may be associated with other behavioral disorders that lead to non-adherence to ART [13]. Alcohol users may also be less likely to seek support from their family, friends, and caregivers, which may lead to non-adherence to ART [36]. Moreover, they may also simultaneously engage in any substance use in order to cope with the treatment side-effects which may also lead to poor treatment compliance [37]. Our results are consistent with the studies conducted in African and Latin American countries, which reported the negative implication of alcohol use on ART adherence [38]. This study provides evidence about the positive association between harmful alcohol drinking and non-adherence to ART in both women and men.

Harmful alcohol drinking had negative effects on the QOL domains of both HIV-positive men and women or simply HIV-positive people. They might have endured poor self-efficacy and not sought appropriate care for opportunistic infections and physical symptoms [39]. Alcohol further increases the risk of the HIV disease progression such as leading to a decrease in CD4 count and an increase in viral load [40]. Eventually, alcohol use might have deteriorated the physical condition of HIV-positive people leading to their poor physical QOL.

In this study, we observed gender differences in the QOL among men and women. Women were more likely to have a poor QOL in respect to social relations and spiritual domains. The lower level of support in women might have had a negative role in those who sought support from their spouse and family [41]. Men were more likely to have a lower QOL in respect to the psychological, level of independence, environment and spiritual domains. Compared to women, Nepalese HIV-positive men might have had problems with multiple substance use and poor engagement in HIV services as reported by the studies in South Africa, where men were more likely to engage in substance use that has negative effect on their use of continuum of care services [42].

This study has some limitations. Social desirability bias may have affected our results as participants self-reported their status regarding missing pills and alcohol use. To minimize this as much as possible the interviews were conducted in private places. Furthermore, we counted consumed pills out of the total prescribed for the previous month. The study districts were selected conveniently to reach the participants from NGOs, and therefore selection bias may limit generalizability of the findings. However, the results can be applied in the settings where participants have similar characteristics. The results might have also been biased by other substance-use disorders and mental health disorders. These factors impact negatively on engagement in HIV services. To minimize this as much as possible we recruited participants from similar settings.

Despite these limitations, this study is novel being the first to examine these issues separately in HIV-positive men and women in Nepal. This study also adds new evidence about the negative association between harmful alcohol drinking, ART adherence, and QOL in both men and women.

## Conclusion

Harmful alcohol drinking was negatively associated with ART adherence and QOL of HIV-positive people in Nepal. When designing services for ART counseling, alcohol-use disorders should be integrated along with psychosocial counseling. Health services providers should pay particular attention to address alcohol-use disorders while providing screening and treatment for HIV-related consequences. Frontline health workers should be provided with training material that are community-based and tailor made so that they can screen for and treat these disorders in their work settings. Additionally, support mechanisms should be established at family and community levels to address alcohol-use disorders, improve ART adherence and enhance the QOL for HIV-positive people. More studies are warranted to investigate causal relationships between harmful alcohol drinking and ART adherence taking into account factors such as depression, stress, and substance-use disorders.

## Paper context

Nepal has been experiencing concentrated epidemics of HIV among most at-risk populations for the past two decades. More than 12,000 out of an estimated 40,000 HIV-positive people are taking anti-retroviral therapy (ART). Among them, harmful alcohol drinking is high in HIV-positive people. Evidence of association between harmful alcohol drinking and ART adherence is limited in Nepal and South Asia. Additionally, the relationship between harmful alcohol drinking and non-adherence by gender is also not well understood. This study investigates association between harmful alcohol drinking (using the AUDIT measure) non-adherence to ART and health-related quality of life in HIV-positive people in Nepal.
